# Characterization of serum proteomic and inflammatory profiling at early stage of iron deficiency in weaned piglets

**DOI:** 10.1016/j.aninu.2024.04.004

**Published:** 2024-04-16

**Authors:** Guang Liu, Lan Li, Shuan Liu, Zhenglin Dong, Jian Zhou, Chengyan Gong, Yulong Yin, Wenjie Tang, Dan Wan

**Affiliations:** a Laboratory of Animal Nutritional Physiology and Metabolic Process, Key Laboratory of Agro-Ecological Processes in Subtropical Region, Institute of Subtropical Agriculture, Chinese Academy of Sciences, Changsha 410125, China; b Hubei Hongshan Laboratory, College of Animal Science and Technology, Huazhong Agricultural University, Wuhan 430070, China; c Beijing Dabeinong Technology Group Co., Ltd., Beijing 100080, China; d University of Chinese Academy of Sciences, Beijing 101408, China; e Animal Breeding and Genetics Key Laboratory of Sichuan Province, Sichuan Animal Science Academy, Chengdu 610066, China; f Livestock and Poultry Biological Products Key Laboratory of Sichuan Province, Sichuan Animtech Feed Co., Ltd., Chengdu 610066, China

**Keywords:** Piglet, Iron deficiency, Proteomics, Inflammatory, Cytokine, Immune response

## Abstract

The objective of this study was to examine the early serum proteomic and inflammatory profiles of weaned piglets subjected to iron deficiency. Twelve healthy piglets (Duroc × Landrace × Large Yorkshire, body weight: 4.96 ± 0.05 kg) were weaned at 21 days of age. Subsequently, these animals were randomly allocated to one of two groups, with six replicates in each group (maintaining a male-to-female ratio of 1:1), the control group (administered 100 mg/kg Fe as FeSO_4_·H_2_O) and L-Fe group (no additional Fe supplementation). The results showed that 42 days after initiating, compared with control group, routine blood analysis revealed a reduction in serum iron content, red blood cell (RBC) count, hemoglobin (HGB) content, hematocrit (HCT), and mean corpuscular volume (MCV) (*P* < 0.05). Subsequent sample analysis indicated a noteworthy decrease in iron deposition in the liver, spleen, and kidneys of piglets fed the L-Fe diet compared with control group (*P* < 0.05). However, final body weight, average daily gain (ADG), average daily feed intake (ADFI), feed conversion ratio, and tissue coefficients were similar between the two groups (*P* > 0.05). During the early stages of iron deficiency, piglets exhibited increased villus height (VH) and the ratio of VH to crypt depth (CD) in the duodenum (*P* < 0.05) and increased expression levels of iron transporters, including duodenal cytochrome (*Cybrd*), divalent metal transport 1 (*DMT1*), and ferritin light chain (*FTL*) (*P* < 0.05). Subsequently, isobaric tags for relative and absolute quantitation (iTRAQ) were used to identify serum proteins. Gene Ontology (GO) analysis of the differentially abundant proteins (DAP) revealed that 24 of the 30 DAP were involved in platelet function, immune response, cellular metabolism, transcription, and protein synthesis. Notably, prothrombin, asporin (*ASPN*), and Rac family small GTPase 3 (*RAC*3) expression was induced, whereas glycoprotein Ib platelet subunit alpha (*GPIbA*) expression was decreased. This was accompanied by a substantial reduction in serum complement 3 (C3) and complement 4 (C4) contents (*P* < 0.05), with elevated the contents of interleukin-1β (IL-1β), interleukin-4 (IL-4), interleukin-6 (IL-6), transforming growth factor-β1 (TGF-β1), and tumor necrosis factor-α (TNF-α) (*P* < 0.05). Our findings underscore the essential role of dietary iron supplementation in maintaining iron homeostasis and modulating inflammatory responses in piglets.

## 1 Introduction

Iron (Fe) is acknowledged as one of the most essential trace elements crucial for animal growth (Jin et al., 2023; Ma et al., 2023). It plays a pivotal role in numerous vital biological functions, encompassing oxygen transport, electron transfer, cellular respiration, and energy metabolism (Rincker et al., 2004). Neonatal piglets are vulnerable to iron deficiency. The National Research Council (NRC, 2012) recommends a standardized dietary contained iron 100 mg/kg for weaned piglets. However, owing to rapid growth, little iron storage and low bioavailability, dietary iron additives might not be sufficient for piglets in livestock production, especially during the weaning period (Eshaghpour et al., 1966; Svoboda and Drabek, 2005). Furthermore, early iron deficiency is often overlooked as it lacks clinical symptoms related to growth and feed consumption. Consequently, iron deficiency remains an important nutritional and metabolic concern in pig production (Dallman, 1987; Rincker et al., 2004).

Iron deficiency is frequently concomitant with various chronic inflammatory diseases (Xiao et al., 2023; Ludwig et al., 2013; Cappellini et al., 2017) that occur due to elevated levels of inflammatory cytokines that regulate hepcidin transcription. The transmembrane function of ferroportin is governed by hepcidin, and increased hepcidin levels promote ferroportin degradation. This degradation, in turn, results in iron deficiency (Hentze et al., 2010; Brasse-Lagnel et al., 2011). Hepcidin expression increases with the activation of inflammatory cytokines, such as interleukin-6 (IL-6) (Armitage et al., 2011). In mice, the administration of 150 or 300 mg of iron chloride solution to iron-deficient mice resulted in a marked decrease in serum contents of interleukin-1α (IL-1α) (Terpilowska and Siwicki, 2009). Therefore, iron deficiency may be accompanied by various inflammatory diseases that can reduce pig performance and even lead to death due to severe inflammation.

The small intestine plays a pivotal role in digestion, absorption, and metabolism of dietary nutrients (Yang et al., 2013; Yin et al., 2023). The structure and function of the small intestine are influenced by changes in the nutritional intake (Pluske et al., 1997). The absorption of dietary ferric iron by duodenal enterocytes occurs primarily through the action of duodenal cytochrome (Cybrd), a ferric reductase that reduces it to ferrous iron. Subsequently, ferrous iron is transported into epithelial cells via divalent metal transport 1 (DMT1) (Lane et al., 2015). Research has indicated that iron has a considerable impact on the morphology of the small intestine. Following ferrous sulfate supplementation, studies have observed a substantial increase in villus height (VH), crypt depth (CD), villus width, and surface area in the small intestine of suckling piglets (Zhou et al., 2021). In mice, iron overload induced by the injection of iron dextran results in atrophy and loss of jejunal villi. Additionally, there is a substantial decrease in jejunal VH and the ratio of VH to CD (VH/CD) (Zhang et al., 2023). In a rat model, overloading with carbonyl iron induces mucosal hypertrophy and alters the expression of transferrin receptors (Oates and Morgan, 1996).

Currently, isobaric tags for relative and absolute quantitation (iTRAQ) are used to measure protein abundance in various samples using MS spectra or specific reporter ions in tandem mass spectrometry (MS/MS) spectra. High-resolution iTRAQ technology is suitable on an increasing number of platforms for the study of proteins and their post-translational modifications in the microbiological, animal, plant, and biomedical fields. Wang and Fang (2016) used iTRAQ technology to perform a proteomic analysis of the endometrial tissues of Meishan and Duroc sows on days 49 and 72 of gestation and identified 4,499 proteins, 45 with upregulated and 69 with downregulated expression. Based on previous studies, the current study was conducted to compare the early effects of iron deficiency on growth performance, iron deposition and absorption, small intestine morphology, and immune cytokine expression in weaned piglets. Serum proteomic analysis was conducted to reveal the full picture of early changes during iron deficiency. The results of this study will provide a basis for the regulation of iron homeostasis, immune responses, and intestinal development in weaned piglets at the early stages of iron deficiency.

## 2 Materials and methods

### 2.1 Animal ethics statement

The experimental procedures were approved by the Protocol Management and Review Committee of the Institute of Subtropical Agriculture, Chinese Academy of Science (No. 20200628), and conducted according to the Institute of Subtropical Agriculture guidelines on Animal Care (Changsha, China).

### 2.2 Animals and experimental treatments

Twelve healthy, weaned piglets (Duroc × Landrace × Large Yorkshire, body weight: 4.96 ± 0.05 kg, weaned at 21 days of age) were selected for the experiment. These animals were randomly assigned to receive one of two groups with six replicates (male: female ratio 1:1) for each group: 1) control group (100 mg/kg Fe as FeSO_4_·H_2_O); 2) L-Fe group (no additional Fe was added). The piglets were fed their respective diets three times a day at 08:00, 12:00, and 20:00 to ensure ad libitum feeding for 42 d. Diets were formulated to meet the nutrient requirements (Table 1) recommended by the National Research Council (NRC, 2012) for weaned piglets during the 42 d of the experimental period, all piglets were individually housed in temperature-controlled, stainless-steel metabolism pens (25 ± 2 °C), allowing free access to drinking water within each 12-h light/dark cycle.

**Table 1:** Composition and nutrient levels of the basal diet (as-fed basis, %).

Item	Content
Corn	24.48
Extruded corn	35.00
Casein	17.20
Corn starch	8.30
Lactose	5.00
Glucose	3.00
Thr	0.12
Trp	0.04
Met	0.23
Lys	0.15
CaCO_3_	0.73
CaHPO_4_	1.67
NaCl	1.00
Antioxidant	0.08
Citric acid	1.50
Antiseptic	0.50
Permix^1^	1.00
Total	100.00
**Calculated nutrient level**^2^	
DE, kcal/kg	3,400
DM	91.13
CP	20.09
EE	2.08
**Measured nutrient levels**^3^	
DM	90.56
CP	19.70
EE	1.95
Fe, mg/kg	29.04

DE = digestive energy; DM = dry matter; CP = crude protein; EE = ether extract.

1 Supplied the following per kilogram of the diet: vitamin A 2,200 IU, vitamin D_3_ 220 IU, vitamin E 16 IU, vitamin K 0.5 mg, vitamin B_1_ 1.5 mg, vitamin B_2_ 4.0 mg, vitamin B_6_ 3.0 g, vitamin B_12_ 0.02 mg, pantothenate 12 mg, nicotinic acid 30 mg, Cu 6 mg, Zn 100 mg, Mn 4 mg, Se 0.3 mg, I 0.14 mg, ZnO 300 mg, colistin 40 mg, 50% olaquindox 20 mg, cefetamet pivoxile hydrochloride 20 mg.

2 Based on NRC (2012) value of ingredients, calculation of DE, DM, CP and EE in the diet.

3 Measured values of DM, CP, EE and Fe in the diet.

### 2.3 Sample collection

On day 42, blood was collected via jugular vein puncture, and 10 mL of blood was collected in a blood collection vessel containing an anticoagulant (EDTA-Na2) for routine blood examination. Blood vessels without anticoagulants were filled with 10 mL of blood, stored at 37 °C for 30 min; hemolytic samples were discarded and centrifuged at 37 °C at 1,200 × *g* for 15 min. The supernatant was collected and stored at −20 °C until biochemical analysis (*n* = 6). Piglets were weighed and one piglet from each replicate was euthanized under prior anesthesia using sodium pentobarbital (40 mg/kg BW) (Ren et al., 2014). The liver, spleen, kidney, and heart were weighed and collected for iron content analysis. Middle sections of the duodenum, jejunum, and ileum were collected for hematoxylin and eosin (HE) staining and histomorphometry. Other pieces of duodenum, jejunum, and ileum samples were placed in liquid nitrogen immediately and stored at −80 °C for latter mRNA analysis.

### 2.4 Analysis of the content of conventional nutrients in samples

All experimental diets were determined in duplicate for dry matter (DM, method 934.01), crude protein (CP, method 990.03), and ether extract (EE, method 920.39), based on the Association of Official Analytical Chemists (AOAC, 2006). The content of iron in the diet and in the liver, heart, spleen, and kidney was analyzed using an inductively coupled plasma emission spectrometer (ICP, 720ES, Agilent, USA) according to a previously published protocol (Liu et al., 2018). Specifically, the DM was measured by weighing 2.00 ± 0.05 g of the sample using an aluminum box with lid and placing the sample in an oven at 100 °C for 5 h until constant weight; the DM content was calculated according to the weight change. After digestion of the dried samples according to method 990.03, the nitrogen content of the samples was determined using a rapid N analyzer (VAP450, Gerhardt, Germany) to calculate the CP content. The EE content of the sample (1.00 ± 0.05 g) was repeatedly extracted with ether to dissolve the fat and lipid substances (Sinopharm, China), and the sample was weighed after extraction. The samples were weighed (1.00 ± 0.05 g) and subjected to digestion under high-temperature conditions using a mixture of nitric acid (Sinopharm, China) and perchloric acid (Sinopharm, China) (v/v, 4/1). After the acids were volatilized, all the samples were filtered and diluted to 10 mL prior to ICP analysis.

### 2.5 Growth performance and organ coefficients

Initial and final body weights of weaned piglets were weighed and recorded at the beginning and end of the trial, and feed intake was weighed and recorded daily. The calculation of organ coefficients of heart, liver, kidney and spleen refers to the method of (Huang et al., 2010):

Organ coefficient (g/kg) = weight of organ (g)/weight of weaned piglet (kg).

The measurement and calculation methods for average daily gain (ADG), average daily feed intake (ADFI), and feed conversion ratio:

ADG (kg/d) = (final body weight − initial body weight)/days on test;

ADFI (kg/d) = total feed intake/days on test;

Feed conversion ratio = total feed intake (kg)/total weight gain (kg).

### 2.6 Blood cell analysis and serum biochemical indices

Hematological indices, including hemoglobin (HGB) content, were analyzed using an automated hematology analyzer (Sysmex KX-21 Hematology Analyzer, Kobe, Japan). An automated biochemistry analyzer (Cobas C311, Roche, Switzerland) was used to analyze serum samples for serum iron, immunoglobulin M (IgM), immunoglobulin G (IgG), complement 3 (C3) and complement 4 (C4) contents. Biochemical kits were purchased from Roche (Shanghai, China).

### 2.7 Serum protein extraction and labeling

A Proteominer kit (Bio-Rad, USA) was used to remove the high abundance of protein in the two groups of piglet serum samples. The protein concentration of each sample was determined using Bradford's method (Datta et al., 2014). Then, 100 μg of protein was extracted accurately from each sample for reductive alkylation and subsequent trypsin digestion at a ratio of protein/enzyme of 20:1. The peptides were then drained using a vacuum centrifugal pump, and the peptides were re-solubilized with 0.5 mol/L triethanolamine borate (TEAB); each set of peptides was labeled with different iTRAQ tags according to the manual.

### 2.8 LC–MS/MS analyses

The samples were separated in the liquid phase using a liquid-phase system (Shimadzu LC-20AB, Shimadzu, Japan), and the separation column was a PolySULFOETHYL SCX column (2.1 mm × 100 mm). The labeled and dried, mixed peptides were desalted using a Waters Sep-Pak Cartridge, dried, and re-solubilized in buffer A (10 mmol/L KH_2_PO_4_ in 25% acetonitrile, pH 2.8). After the column was loaded, a gradient elution was performed at a rate of 0.2 mL/min, buffer A was eluted for 10 min, followed by 0 to 35% buffer B (10 mmol/L KH_2_PO_4_, 350 mmol/L KCl in 25% acetonitrile, pH 2.8) for 30 min, and then 35% to 80% buffer B was gradually mixed in for 2 min. The entire elution process was monitored at an absorbance of 214 nm and 30 fractions were obtained after screening. Each fraction was desalted separately using a Strata X desalting column and then freeze-dried.

The dried SCX fractions were resolubilized and subjected to two consecutive liquid quality analyses. The liquid-phase system combined with the mass spectrometer was a HPLC system (20AD, Shimadzu, Japan) and consisted of a Micromass C_18_ column (5 μm, 300 Å, 0.1 mm × 15 mm). The mobile phases used were liquid A (water:acetonitrile:formic acid = 98:2:0.1) and liquid D (water:acetonitrile:formic acid = 2:98:0.1), with an appropriate amount of the calibration solution (Thermo Fisher Scientific, USA). After peptide adsorption and desalting, the samples were processed using a TripleTOF 5600 (AB SCIEX, Concord, ON, Canada) with a Nanospray III source (AB SCIEX, Concord, ON, Canada) serving as the ion source and a quartz-drawn spray needle (New Objectives, Woburn, MA, USA) as the emitter for MS/MS analysis. During data collection, the machine parameters were configured as follows: ion source spray voltage of 2.5 kV, nitrogen pressure of 30 psi (14.5 psi = 1 bar), spray pressure of 15 psi, and spray interface temperature of 150 °C; scanning mode was reflection mode. Ions from 2^+^ to 5^+^ were accumulated for 250 ms, with the first 50 ions accumulating more than 120 scores per second being scanned, and 2.8 s constituting a cycle. The transmission window of the second quadrupole (Q2) was set to 100 Da for 100%; the frequency of pulsed RF electricity was 11 kHz, and the detection frequency of the detector was 40 GHz. The particle signal of each scan was recorded in four channels for a total of four times and then merged into data. For iTRAQ, the ion fragmentation energy was set to 35 ± 5 eV; parent ion dynamic exclusion was set to 18 s, the same parent ion did not undergo fragmentation more than twice.

### 2.9 Bioinformatics analysis

The mass spectrometry data obtained were retrieved using Mascot (Matrix, USA) and then analyzed using Scaffold (Proteome Software, UAS) for relative quantification. Proteins were regarded as differentially abundant proteins (DAP) when the multiplicity of difference in protein abundance reached more than 1.2-fold and the *P*-value was less than 0.05 using a statistical test. Protein annotation and classification were performed using DAVID (Sherman et al., 2022), in which biological processes, cellular components, and molecular function annotations from Gene Ontology (GO) were selected to classify proteins.

### 2.10 Cytokine microarray

Reagent kits (Raybiotech, USA) were used for the cytokine microarray technique to detect differences in the serum cytokine content interleukin-1β (IL-1β), interleukin-4 (IL-4), interleukin-6 (IL-6), interleukin-12 (IL-12), granulocyte-macrophage colony-stimulating factor (GM-CSF), transforming growth factor-β1 (TGF-β1) and tumor necrosis factor-α (TNF-α) between the control and L-Fe groups.

### 2.11 Quantitative real time-PCR (qRT-PCR)

Total RNA was isolated from duodenum, jejunum, and ileum samples frozen in liquid nitrogen using TRIzol reagent (Invitrogen, Carlsbad, CA, USA) and treated with Dnase I (Invitrogen) according to the manufacturer's instructions. The primers were designed using Primer 5.0 (Table 2). The LightCycler 480 Instrument (Applied Biosystems, Carlsbad, CA) was used to quantify mRNA expression. Glyceraldehyde-3-phosphate dehydrogenase (*GAPDH*) was used as the reference gene to normalize the expression of iron homeostasis-related genes. Gene relative expression levels were calculated as previously reported methodology (Zhou et al., 2017).

**Table 2:** Primers used in this study.

Gene	Primer sequences (5′ to 3′)	GenBank No.
*GAPDH*	F: GGGCATGAACCATGAGAAGT R: AGCACCAGTAGAAGCAGGGA	NM_001206359
*Cybrd*	F: AGATTGGCCCTGGAGACTGA R: CAAGGAAGCCTTGGGTGAAG	XM_005671927.3
*DMT1*	F: AGGATCTAGGGCATGTGGTG R: CCACAGTCCAGGAAGGACAT	NM_001128440.1
*FPN*	F: TGTCCACTGGGTGTCTGTGT R: TGTTCATTCACTTCCTCTCTTTTC	XM_003483701.4
*IPR1*	F: CTGTGGGAATGTTTCGGGAT R: CCACTGCAGCAAGGCACTAC	XM_003357729.3
*IPR2*	F: TGGTCATTGCTGCCGTTATC R: TGTAACCATCCCACTGCCTG	NM_001167781.1
*TFRC*	F: GTTTAGCGCAGGAGTGAGGC R: TGGGACAAGCAACAGAGGAA	NM_214001.1
*FTL*	F: AAAACCCAGGACGCTATGGA R: CCAGGAAGTGGTTCTCCAGG	NM_001244131.1

*GAPDH* = glyceraldehyde-3-phosphate dehydrogenase; *Cybrd* = duodenal cytochrome; *DMT1* = divalent metal transport 1; *FPN* = ferroportin; *TFRC* = transferrin receptor; *FTL* = ferritin light chains; *IPR1* = iron regulatory protein 1; *IPR2* = iron regulatory protein 2.

### 2.12 Histopathology

The duodenum, jejunum, and ileum were incubated in 4% neutral-buffered 10% formalin until paraffin embedding. After fixation, paraffin embedding was performed and sections (at least three layers; thickness of 5 μm) were stained with hematoxylin and eosin. Images were acquired using a laser scanning confocal microscope (LSM880, Zeiss, Germany), and the VH and CD were measured (Liu et al., 2023).

### 2.13 Statistical analysis

The results were analyzed using a completely randomized study design. None of the animals exhibited growth arrest during the trial. Therefore, all animal data were included. Given the similar growth rates of the male and female piglets in the feeding trial, sex effects were not considered in this study.

Data are presented as the mean values. All statistical analyses were performed using SPSS 26.0 software (IBM Corp., Armonk, NY, USA). Differences between the treatments were evaluated using a simple *t*-test. For the analysis, the dietary treatment was considered a fixed effect, and the animal was considered a randomized factor. *P* < 0.05 were considered statistically significant.

## 3 Results

### 3.1 Growth performance and organ coefficients of weaned piglets

The effects of a low iron diet on body weight, feed intake, feed conversion ratio, and organ coefficients are shown in Table 3. Compared with the control group, ADG, ADFI, feed conversion ratio, organ weight, and organ coefficients of the heart, liver, kidney, and spleen were not affected by the low-iron diet during the experimental period (*P* > 0.05).

**Table 3:** Effects of low iron diet on growth performance and organ coefficients of weaned pigs (*n* = 6).

Item	Control group	L-Fe group	SEM	*P*-value
Initial body weight, kg	4.95	4.97	0.051	0.867
Final body weight, kg	16.45	16.08	0.524	0.744
Average daily gain, kg/d	0.27	0.27	0.004	0.333
Average daily feed intake, kg/d	0.75	0.76	0.017	0.722
Feed conversion ratio	2.73	2.87	0.082	0.427
Heart weight, g	80.62	78.15	3.306	0.728
Heart organ coefficient, g/kg	4.91	4.83	0.095	0.669
Liver weight, g	518.00	485.82	23.480	0.519
Liver organ coefficient, g/kg	31.53	29.99	0.735	0.317
Spleen weight, g	47.30	41.42	3.401	0.413
Spleen organ coefficient, g/kg	2.89	2.57	0.198	0.439
Kidney weight, g	103.02	100.68	5.775	0.851
Kidney organ coefficient, g/kg	6.26	6.19	0.238	0.891

Basal diet analyzed 29.04 mg/kg Fe DM. Supplemental Fe was 0 mg/kg (L-Fe group) and 100 mg/kg (control group).

### 3.2 Hematological parameters and iron content in tissues

Compared to the control group, red blood cell (RBC) count, hemoglobin (HGB) content, hematocrit (HCT), mean corpuscular volume (MCV), and serum iron content were reduced (*P* < 0.05) in the iron-deficient piglets (Table 4). Compared to the control group, the iron contents in the liver, kidney, and heart were lower than those in the L-Fe group (*P* < 0.05) (Table 5).

**Table 4:** Effects of low iron diet on blood state of weaned piglets (*n* = 6).

Item	Control group	L-Fe group	SEM	*P*-value
RBC, ×10^12^/L	5.87	4.89	0.149	<0.001
HGB, g/L	124.00	102.50	3.250	<0.001
HCT, %	0.39	0.31	0.012	<0.001
MCV, fL	66.27	62.50	0.639	0.001
Serum iron, μg/L	1,560.00	690.00	179.344	0.007

RBC = red blood cell; HGB = hemoglobin; HCT = hematocrit; MCV = mean corpuscular volume.

Basal diet analyzed 29.04 mg/kg Fe DM. Supplemental Fe was 0 mg/kg (L-Fe group) and 100 mg/kg (control group).

**Table 5:** Effects of low iron diet on tissue iron contents (mg/kg) in liver, kidney, heart, spleen of the weaned piglets (*n*=6).

Item	Control group	L-Fe group	SEM	*P*-value
Liver iron	64.76	33.14	7.228	0.020
Kidney iron	33.06	22.07	2.445	0.016
Heart iron	39.71	32.21	1.683	0.017
Spleen iron	140.64	77.64	14.327	0.091

Basal diet analyzed 29.04 mg/kg Fe DM. Supplemental Fe was 0 mg/kg (L-Fe group) and 100 mg/kg (control group).

### 3.3 Gene relative expression in iron homeostasis

**Fig. 1:**
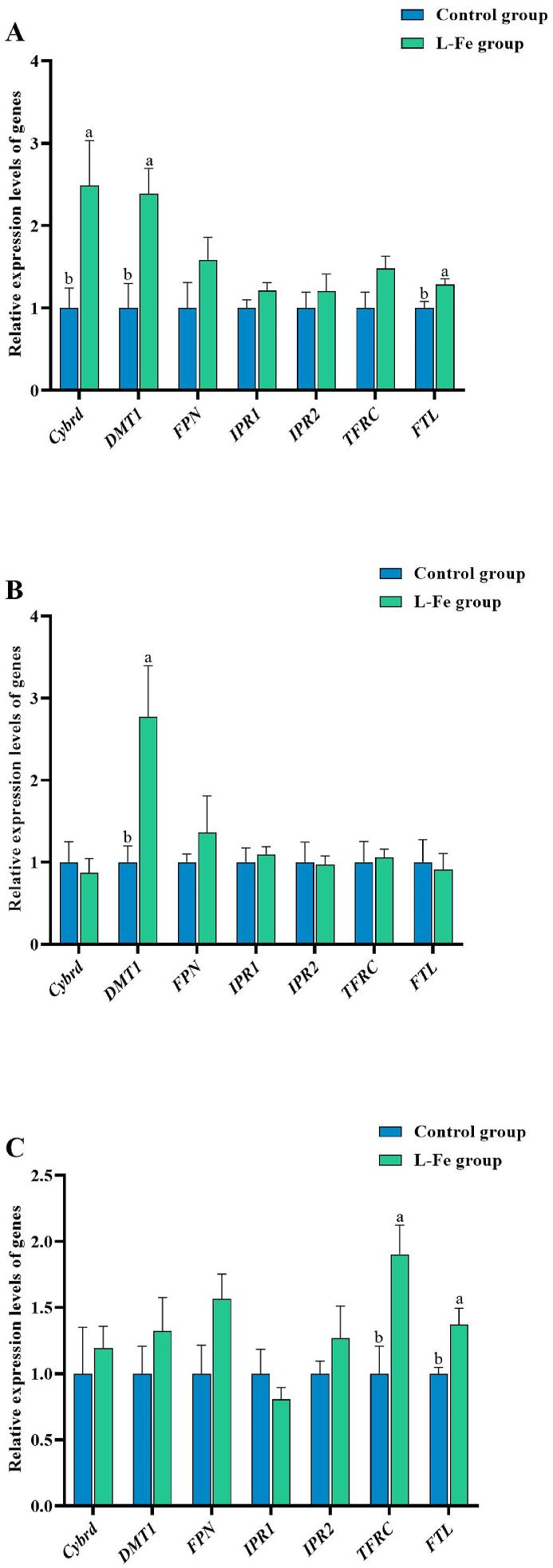
Effects of low iron diet on the relative expression levels of genes related to iron absorption in duodenum (A), jejunum (B) and ileum (C) of the weaned piglets (*n* = 6). *Cybrd* = duodenal cytochrome; *DMT1* = divalent metal transport 1; *FPN* = ferroportin; *IPR1* = iron regulatory protein 1; *IPR2* = iron regulatory protein 2; *TFRC* = transferrin receptor; *FTL* = ferritin light chains. Supplemental Fe was 100 mg/kg (control group) and 0 mg/kg (L-Fe group). Values are means, with standard errors represented by vertical bars. Different letters represent significant difference (*P* < 0.05).

The relative expression levels of *Cybrd*, *DMT1*, ferroportin (*FPN*), iron regulatory protein 1 (*IPR1*), iron regulatory protein 2 (*IPR2*), transferrin receptor (*TFRC*), ferritin light chains *(FTL)* genes in the duodenum (Fig. 1A), jejunum (Fig. 1B), and ileum (Fig. 1C) of all weaned piglets were tested using qRT-PCR. Results showing that the relative expression levels of *Cybrd*, *DMT1*, and *FTL* genes increased in the duodenum of the L-Fe group (*P* < 0.05; Fig. 1A). Moreover, *DMT1* gene relative expression level was higher than that of the control group in the jejunum (*P* < 0.05; Fig. 1B). In addition, the relative expression levels of *TFRC* and *FTL* genes were increased in the ileum of the L-Fe group compared to that in the control group (*P* < 0.05; Fig. 1C).

### 3.4 Histopathology

**Fig. 2:**
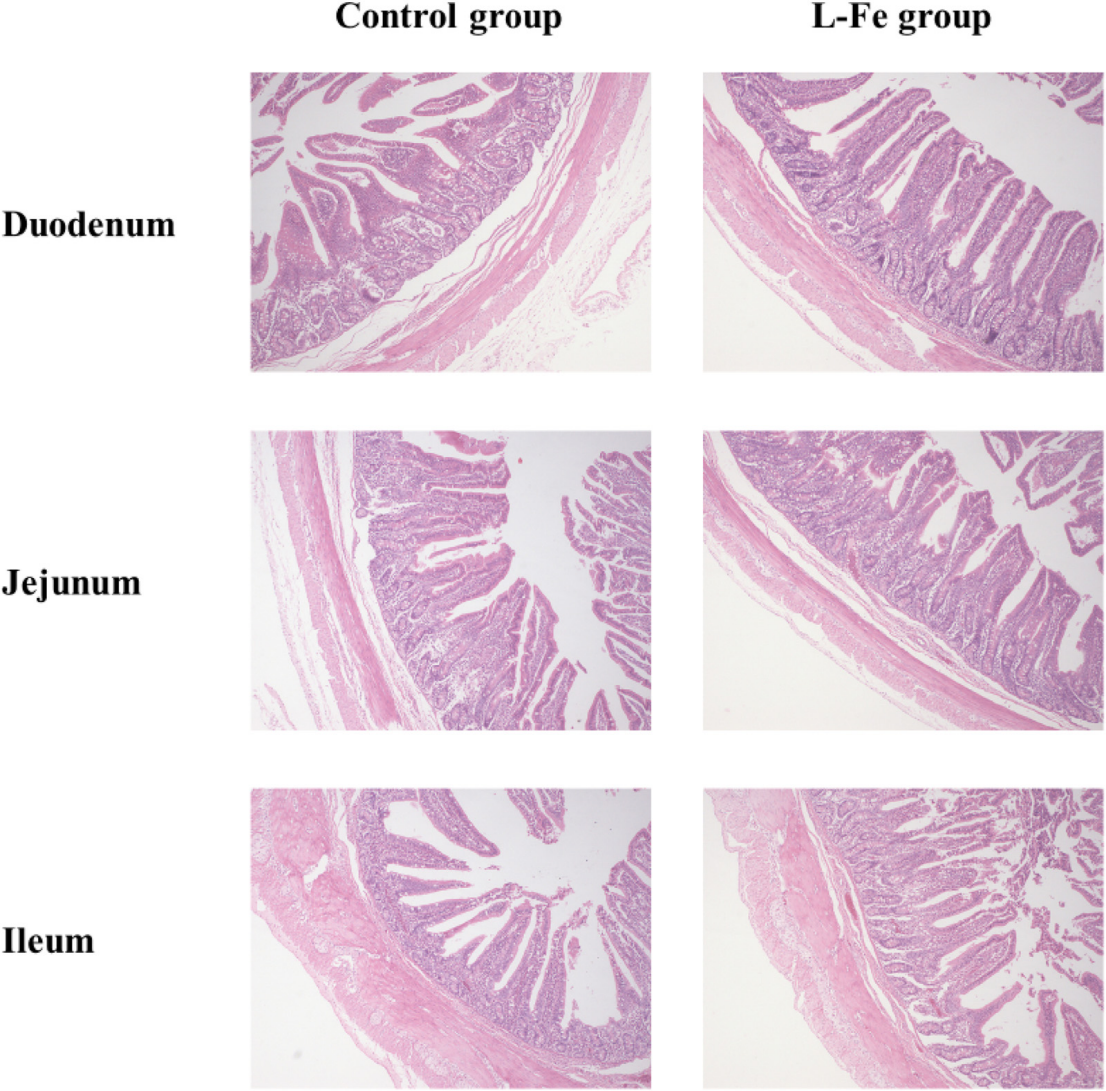
Stained with hematoxylin and eosin (HE) in the duodenum, jejunum and ileum tissues (40 ×). Supplemental Fe was 100 mg/kg (control group) and 0 mg/kg (L-Fe group).

Intestinal VH, CD, and VH/CD are presented in Table 6 and Fig. 2. The VH, CD, and VH/CD of the jejunum and ileum were similar to those of the control group (*P* > 0.05). However, duodenal VH in the L-Fe group was higher than that in the control group (*P* = 0.003). Moreover, the VH/CD of the duodenum was higher in the L-Fe group than in the control group (*P* = 0.002).

**Table 6:** Effects of low iron diet on small intestinal morphology of weaned piglets (*n* = 6).

Item	Control group	L-Fe group	SEM	*P*-value
Duodenum VH, μm	393.09	532.24	27.082	0.003
Duodenum CD, μm	116.47	111.25	2.756	0.368
Duodenum VH/CD	3.37	4.82	0.276	0.002
Jejunum VH, μm	421.49	501.40	28.926	0.181
Jejunum CD, μm	112.03	117.91	3.079	0.369
Jejunum VH/CD	4.30	3.78	0.284	0.387
Ileum VH, μm	414.63	371.63	23.243	0.385
Ileum CD, μm	113.16	97.78	5.028	0.134
Ileum VH/CD	3.92	3.65	0.276	0.642

VH = villus height; CD = crypt depth.

Basal diet analyzed 29.04 mg/kg Fe DM. Supplemental Fe was 0 mg/kg (L-Fe group) and 100 mg/kg (control group).

### 3.5 Serum proteome

**Fig. 3:**
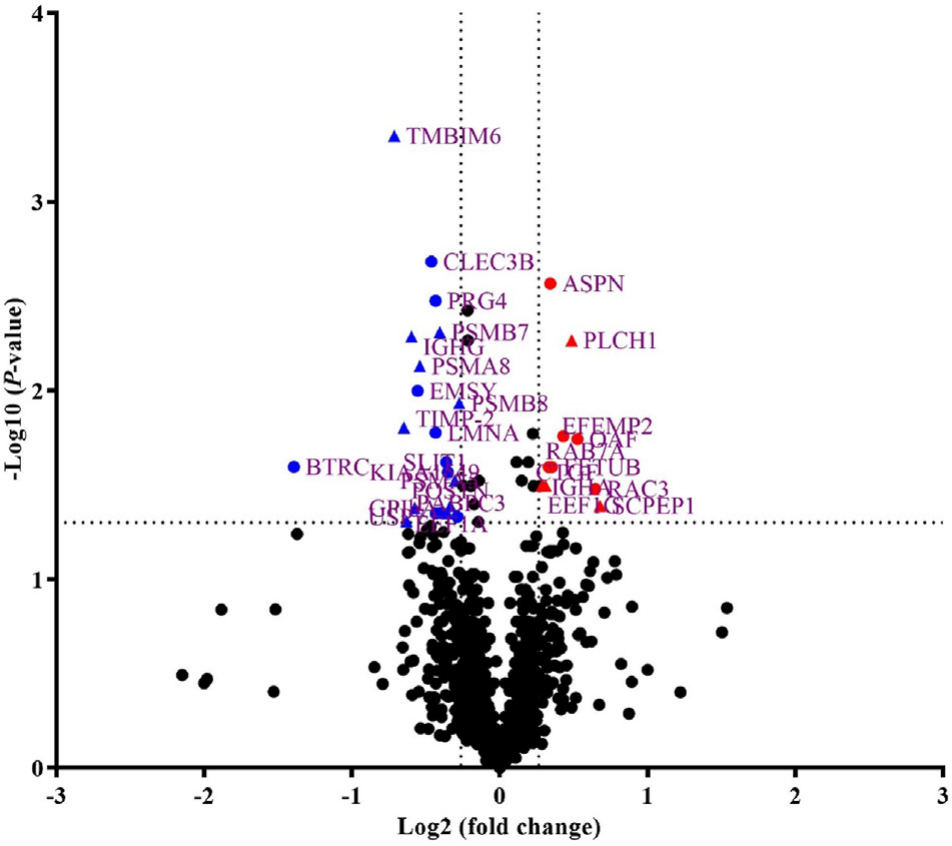
Volcano diagrams constructed from fold changes and *P*-values of serum proteins in low iron diets and control piglets. With log 2 (fold change) as the horizontal coordinate and −log 10 (*P*-value) of the *t*-test significance test *P*-value as the vertical coordinate, changes greater than 1.2-fold and *P* < 0.05 are DAP (red in the graph is significant up-regulation and blue is significant down-regulation). ASPN = asporin; BTRC = beta-transducin repeat containing E3 ubiquitin protein ligase; CLEC3B = C-type lectin domain family 3 member B; CTGF = connective tissue growth factor; EEF1A = eukaryotic translation elongation factor 1 alpha 1; EEF1G = eukaryotic translation elongation factor 1 gamma; EFEMP2 = EGF containing fibulin extracellular matrix protein 2; EMSY = EMSY transcriptional repressor; FETUB = fetuin B; GPIbA = glycoprotein Ib platelet subunit alpha; IGHA = immunoglobulin heavy constant alpha; IGHG = immunoglobulin heavy constant gamma; LMNA = lamin A/C; OAF = out at first homolog; PABPC3 = poly(A) binding protein cytoplasmic 3; PLCH1 = phospholipase C Eta 1; POSTN = periostin; PRG4 = proteoglycan 4; PSMA5 = proteasome 20S subunit alpha 5; PSMA8 = proteasome 20S Subunit alpha 8; PSMB7 = proteasome 20S subunit beta 7; PSMB8 = proteasome 20S subunit beta 8; RAB7A = RAS-related in brain 7A; RAC3 = Rac family small GTPase 3; SCPEP1 = secreted phosphoprotein 1; SLIT1 = slit guidance ligand 1; TIMP-2 = tissue inhibitor of metalloproteinase-2; TMBIM6 = transmembrane BAX inhibitor motif containing 6; USP7 = ubiquitin specific peptidase 7. Supplemental Fe was 100 mg/kg (control group) and 0 mg/kg (L-Fe group).

Among the 1,196 proteins identified by iTRAQ, 43 proteins in the L-Fe group were different from the control group (*P* < 0.05), with a threshold of at least 1.2-fold change in the expression levels of 30 DAP. A volcano plot of DAP (Fig. 3) is shown. Gene Ontology analysis of all DAP revealed that 24 of the 30 DAP found in the serum were involved in platelet function, immune response, cellular metabolism, transcription, and protein synthesis (Table 7, Table 8). Of the proteins, 12 of the 30 DAP were associated with the immune response. The abundance of proteins including R4H4K5, F1SUE4, I3LKU0, and K7ZRK0 was higher; A0SEG9, B6ECP2, F1RS37, I3L5Z3, F1S8U2, L8AXM9, I3LQP7, and C0JPM4 were lower in mildly iron-deficient piglets. Eight of the 30 DAP were related to transcription and protein synthesis, where the abundances of Q29387 and Q0PY11 increased, and the abundances of B6DT15, F2Z557, F2Z5K2, F1SBA5, A5D9J4, and A1XQU1 decreased. Moreover, five of the 30 DAP, including increased O97765, I3LFF0, and F1RSC3, and decreased I3LQP7 and C0JPM4, were associated with cellular metabolism. The proteins (B3STX9 and B6ECP2), which are associated with platelet function, also fluctuated in response to the iron status.

**Table 7:** Fold changes in serum of specific functional differentially abundant proteins in piglets weaned on low iron diets (*n* = 6).

Accession no.	Gene name	Protein name	Annotation	Fold change (L-Fe/control)	*P*-value
Platelet function					
B3STX9	–	Prothrombin	Prothrombin	1.22	0.093
B6ECP2	*GPIbA*	Glycoprotein Ib platelet subunit alpha	Platelet glycoprotein Ib alpha polypeptide	0.67	0.037
Immune response					
A0SEG9	*C9*	Complement component C9	Complement component C9	0.80	0.009
B6ECP2	*GPIbA*	Glycoprotein Ib platelet subunit alpha	Platelet glycoprotein Ib alpha polypeptide	0.67	0.037
F1RS37	*POSTN*	Periostin	Uncharacterized protein	0.76	0.041
R4H4K5	–	Retinoic acid receptor responder protein 2	Chemerin	1.24	0.013
F1SUE4	*ASPN*	Asporin	Uncharacterized protein	1.27	0.003
I3L5Z3	*PRG4*	–	Uncharacterized protein	0.74	0.003
I3LKU0	*RAC3*	Rac family small GTPase 3	Uncharacterized protein	1.56	0.029
F1S8U2	*BTRC*	Beta-transducin repeat containing E3 ubiquitin protein ligase	Uncharacterized protein	0.38	0.031
K7ZRK0	*IGHA*	IgA heavy chian constant region	IgA heavy chain constant region (fragment)	1.24	0.032
L8AXM9	*IGHG*	IgG heavy chain	IgG heavy chain	0.66	0.005
I3LQP7	*TMBIM6*	Bax inhibitor 1	Bax inhibitor 1	0.61	<0.001
C0JPM4	TIMP-2	Metalloproteinase inhibitor-2	Tissue inhibitor of metalloproteases 2	0.64	0.019
Cell metabolism					
I3LQP7	*TMBIM6*	Bax inhibitor 1	Bax inhibitor 1	0.61	<0.001
C0JPM4	*TIMP-2*	Metalloproteinase inhibitor-2	Tissue inhibitor of metalloproteases-2	0.64	0.019
O97765	*CTGF*	Connective tissue growth factor	Connective tissue growth factor	1.22	0.033
I3LFF0	*PLCH1*	Phosphoinositide phospholipase C1	Phosphoinositide phospholipase C1	1.40	0.005
F1RSC3	*SCPEP1*	Carboxypeptidase 1	Carboxypeptidase 1	1.60	0.041
Transcription and protein syntheses					
B6DT15	*USP7*	Ubiquitin carboxyl-terminal hydrolase 7	Ubiquitin-specific peptidase 7	0.65	0.050
F2Z557	*PABPC3*	Polyadenylate-binding protein 3	Polyadenylate-binding protein 3	0.78	0.044
F2Z5K2	*PSMA5*	Proteasome subunit alpha type5	Proteasome subunit alpha type5	0.81	0.034
F1SBA5	*PSMA8*	Proteasome subunit alpha type 8	Proteasome subunit alpha type8	0.69	0.007
A5D9J4	*PSMB8*	Proteasome subunit beta 8	Proteasome subunit beta type8	0.83	0.011
A1XQU1	*PSMB7*	Proteasome subunit beta type 7	Proteasome subunit beta type7	0.75	0.005
Q29387	*EEF1G*	Elongation factor 1-gamma	Elongation factor 1-gamma	1.21	0.028
Q0PY11	*EEF1A*	Elongation factor 1-alpha	Elongation factor 1-alpha	1.35	0.023

**Table 8:** Gene Ontology annotation of differentially abundant proteins involved in immune responses.

Accession no.	Gene name	GO annotation	
B6ECP2	*GPIbA*	GO:0046426	Negative regulation of JAK-STAT cascade
		GO:0019221	Cytokine-mediated signaling pathway
F1SUE4	*ASPN*	GO:0046426	Negative regulation of JAK-STAT cascade
		GO:0019221	Cytokine-mediated signaling pathway
F1RS37	*POSTN*	GO:0008593	Regulation of Notch signaling pathway
F1S8U2	*BTRC*	GO:0043122	Regulation of I-kappaB kinase/NF-kappaB signaling
A0SEG9	–	GO:0045087	Innate immune response
I3L5Z3	*PRG4*	GO:0006955	Immune response
R4H4K5	–	GO:0006954	Inflammatory response
C0JPM4	*TIMP-2*	GO:0034097	Response to cytokine
I3LKU0	*RAC3*	GO:0071593	Lymphocyte aggregation
		GO:0042129	Regulation of T cell proliferation
I3LQP7	*TMBIM6*	GO:0051025	Negative regulation of immunoglobulin secretion

*GPIbA* = glycoprotein Ib platelet subunit alpha; *ASPN* = asporin; *POSTN* = periostin; *BTRC* = beta-transducin repeat containing E3 ubiquitin protein ligase; *PRG4* = proteoglycan 4; *TIMP-2* = tissue inhibitor of metalloproteinase-2; *RAC3* = Rac family small GTPase 3; *TMBIM6* = transmembrane BAX inhibitor motif containing 6; JAK-STAT = Janus kinase/signal transducer and activator of transcription; NF-kappaB = nuclear factor-kappa B.

### 3.6 Serum immunity and cytokines

**Fig. 4:**
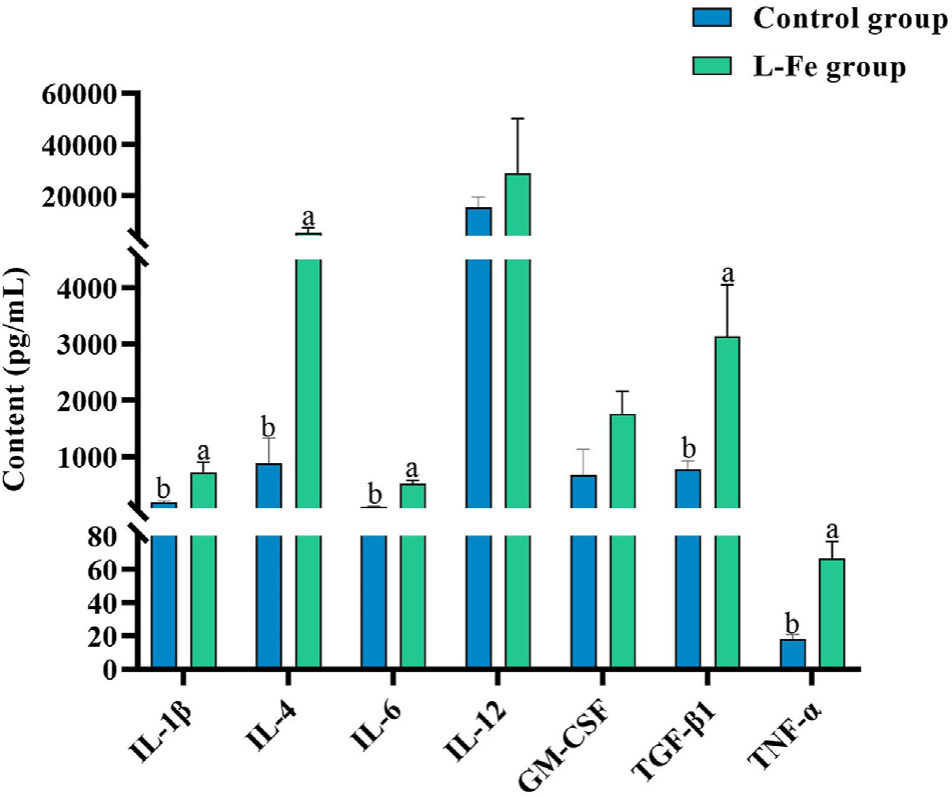
Effects of low iron diet on contents of inflammatory cytokines in the serum of weaned piglets (*n* = 6). IL-1β = interleukin-1β; IL-4 = interleukin-4; IL-6 = interleukin-6; IL-12 = interleukin-12; GM-CSF = granulocyte-macrophage colony-stimulating factor; TGF-β1 = transforming growth factor-β1; TNF-α = tumor necrosis factor-α. Supplemental Fe was 100 mg/kg (control group), 0 mg/kg (L-Fe group). Values are means, with standard errors represented by vertical bars. Different letters represent significant difference (*P* < 0.05).

There was no marked change in IgM or IgG contents in the serum of piglets in the L-Fe group compared to those in the control group (*P* *>* 0.05; Table 9). However, C3 and C4 contents in the L-Fe group were lower than those in the control group (*P* < 0.05). To determine whether iron deficiency leads to the development of inflammation, the contents of IL-1β, IL-4, IL-6, IL-12, GM-CSF, TGF-β1, and TNF-α in the serum of weaned piglets in each group were measured using a cytokine microarray (Fig. 4). The contents of IL-1β, IL-4, IL-6, TGF-β1, and TNF-α in the serum of piglets in the L-Fe group were higher than those in the control group (*P* < 0.05), whereas the differences in the contents of IL-12 and GM-CSF were similar (*P* > 0.05).

**Table 9:** Effects of low iron diet on the contents (mg/mL) of IgM, IgG, C3 and C4 in serum of weaned piglets (*n*=6).

Item	Control group	L-Fe group	SEM	*P*-value
IgM	0.31	0.26	0.032	0.405
IgG	1.56	1.54	0.057	0.846
C3	0.13	0.08	0.008	0.002
C4	0.05	0.04	0.003	0.029

IgM = immunoglobulin M; IgG = immunoglobulin G; C3 = complement protein 3; C4 = complement protein 4.

Basal diet analyzed 29.04 mg/kg Fe DM. Supplemental Fe was 0 mg/kg (L-Fe group) and 100 mg/kg (control group).

## 4 Discussion

The effects of early iron deficiency on iron homeostasis, immune response, and intestinal development in weaned piglets were investigated in the present study. Iron content in tissues is regarded as a reliable response criterion for evaluating mineral status (Feng et al., 2009). In the present study, the iron content in the liver, spleen, kidney, and heart decreased substantially, indicating that the weaned piglets were fed an iron-deficient diet, likely in an iron-deficient state. Iron deficiency may lead to decreased growth performance, and growth performance and intestinal barrier function have been reported to be impaired in iron-deficient weaned piglets (Hansen et al., 2009; Perri et al., 2016). However, our data indicated that the changes in growth performance, organ weight, and organ coefficient of weaned piglets were not significant. However, iron metabolism was substantially altered, suggesting that these piglets were at an early stage of iron deficiency or had a mild iron deficiency.

Iron is an essential element in the body and the most basic material for the synthesis of hemoglobin and erythrocytes. Iron deficiency affects the synthesis of hemoglobin, which in turn reduces the hemoglobin content and the oxygen-carrying capacity of erythrocytes, leading to iron deficiency anemia (Brugnara et al., 1999). In this study, after feeding an iron-deficient diet for 42 d, the serum iron contents in the L-Fe group were significantly lower, and the HGB content in the L-Fe group was reduced to 102.50 g/L, which was substantially lower than that in the control group (124.00 g/L). Since pigs with HGB content below 100 g/L are classified as mildly anemic (Kim et al., 2018), the L-Fe group was close to mildly iron-deficient. This is consistent with the results of previous studies, in which HGB content and MCV increased with increasing dietary iron content (Feng et al., 2007; Dong et al., 2023; Liu et al., 2023).

To explore the reaction of the small intestine to an iron-deficient diet, the expression of the genes, *Cybrd*, *DMT1*, *FPN*, *IPR1*, *IPR2*, *TFRC* and *FTL*, which are involved in iron absorption and transportation in the small intestine, was determined. Cybrids are the primary mammalian transplasma ferric reductases. In the duodenum, ferric iron is reduced by cybard and transported by DMT1 (Lane et al., 2015). TFRC transports transferrin-binding iron. Consistent with previous reports in other animals, the expression of *Cybrd* and *DMT1* genes was higher in the duodenum of piglets in the L-Fe group, suggesting an increased capability for the absorption and transportation of iron to meet iron requirements (Fcollins, 2006; Hansen et al., 2009), but there was no significant difference on expression of *TFRC* gene. Ferroportin, a critical protein for iron transportation from duodenal enterocytes to the blood, is regulated by hepcidin (Donovan et al., 2005). In our study, there was no significant difference in *FPN* expression in mildly iron-deficient piglets, whereas it has been documented that targeted deletion of *FPN* in macrophages results in a relatively mild iron deficiency in a rat model (Zhang et al., 2011). However, the expression of *FTL* gene was markedly elevated in the duodenum and ileum of the L-Fe group. Ferritin light chains is a clear marker of coronary atherosclerosis (You et al., 2003) and its expression is increased in glioblastomas (Wu et al., 2016). Unexpectedly, the expression levels of *IRP1* and *IRP2* genes were similar between the control and L-Fe groups. The *IPR1*/*IPR2* had been proven to be involved in regulating the expression of iron metabolism- and transport-related proteins to optimize cellular iron availability at the post-transcriptional level (Rouault, 2006). However, they are primarily located in the liver. Thus, *IPR1*/*IPR2* may not participate in exogenous iron absorption in the small intestine. Thus, at the early stages of iron deficiency, it may alter the expression of iron absorption-related genes in the small intestine, resulting in improved digestion and absorption of iron. The duodenum is the primary organ responsible for iron absorption in the small intestine (Frazer and Anderson, 2005; Ganz and Nemeth, 2006). In the current study, the VH/CD in the duodenum of piglets in the L-Fe group increased, which may also help improve iron bioavailability from food, thereby reducing the symptoms of iron deficiency in piglets.

Immunoglobulins are important components of the blood. In previous studies, an increased risk of immune deficiency and infection was observed in the presence of iron deficiency (Jonker and van Hensbroek, 2014). A randomized trial follow-up study of Kenyan infants found that infants who were supplemented with iron at the time of vaccination showed higher IgG content, seroconversion, and IgG avidity than those who did not (Stoffel et al., 2020). Contrary to a previous study (Sadeghian, 2010), our results in piglets showed that serum immunoglobulin content did not change during the early stages of iron deficiency. However, the contents of the important complement components, C3 and C4, which participate in the body's immune response through various complement activation pathways and contribute to early defense against infection, were substantially lower than those in the control group (Galan et al., 1988). Therefore, iron deficiency may result in immune dysfunction via complement activation pathways and exacerbate the inflammatory responses (Rayes et al., 2018).

In the present study, we demonstrated that iron deficiency led to an enhanced pro-inflammatory response, resulting in a marked increase in serum contents of the inflammatory cytokines, IL-1β, IL-4, IL-6, TGF-β1, and TNF-α and a trend towards increased contents of IL-12 and GM-CSF. The higher inflammatory response in piglets without additional iron in the diet in this study is consistent with previous studies in which low iron status led to increased pro-inflammatory cytokines (including IL-1β, IL-6, and TNF-α) in iron deficiency anemia (Ferrucci et al., 2010; Pu et al., 2015). Therefore, according to our research, the immunization of piglets in a state of iron deficiency increases the risk of early infection during production.

The iTRAQ analysis indicated that the abundance of differentially expressed proteins was closely associated with the expression of common inflammatory mediators such as IL-6, IL-8, and TNF-α in the serum of the L-Fe group of piglets. In the L-Fe group, the serum expression of *ASPN* and *RAC3* increased 1.27- and 1.56-fold, respectively, whereas *GPIbA* expression decreased 0.67-fold. Asporin has been shown to strongly inhibit apoptosis, promote growth in gastric cancer cells, and selectively promote LEF1 binding to activate the promoters of PTGS2, IL-6, and WISP1 to facilitate their transcription (Zhang et al., 2021). Rac family small GTPase 3 regulates the secretion of matrix metalloproteinase-9 (MMP-9), IL-6, IL-8, and growth-related oncogene (GRO), as well as resistance to TNF-induced apoptosis (Gest et al., 2013). Glycoprotein Ib platelet subunit alpha plays a key role in hemostasis and has long been recognized as a key gene that mediates platelet coagulation. These previous findings could help to explain the synchronous changes in inflammatory cytokines (IL-1β, IL-4, IL-6, TGF-β1, and TNF-α) and the ASPN, RAC3, and GPIbA abundance in the present study. According to changes in prothrombin and GPIbA, piglets at an early stage of iron deficiency have increased compensatory hemagglutination, which may result in reduced bleeding after trauma (Lanza, 2006).

## 5 Conclusion

In conclusion, although growth performance remained unaffected during the early stages of iron deficiency, there were substantial reductions in serum iron content, RBC counts, HGB content, HCT, and MCV. The height of the duodenal villi increased along with an upregulation in the expression of genes associated with iron absorption to facilitate increased iron uptake by the body. However, alterations in several serum proteins indicate that iron deficiency can lead to increased pro-inflammatory responses and can affect immune function. These results suggest that early iron deficiency activates immune responses and is detrimental to the intestinal health of piglets.

## Author contributions

**Guang Liu:** Conceptualization, Writing-Original draft preparation, Software. **Lan Li**: Data curation, Methodology. **Shuan Liu:** Project administration, Data curation. **Zhenglin Dong:** Visualization. **Jian Zhou:** Software. **Chengyan Gong**: Project administration, Data curation. **Yulong Yin:** Writing-Reviewing and Editing, Supervision. **Wenjie Tang:** Funding acquisition, Writing-Reviewing and Editing. **Dan Wan:** Funding acquisition, Writing-Reviewing and Editing.

## Declaration of competing interest

We declare that we have no financial and personal relationships with other people or organizations that can inappropriately influence our work, and there is no professional or other personal interest of any nature or kind in any product, service and/or company that could be construed as influencing the content of this paper.
